# A mixed methods study of Canadian adolescents’ perceptions of health

**DOI:** 10.3402/qhw.v11.32891

**Published:** 2016-10-13

**Authors:** Valerie Michaelson, William Pickett, Eleanor Vandemeer, Brian Taylor, Colleen Davison

**Affiliations:** 1 School of Religion, Queen's University, Kingston, Ontario, Canada; 2 Department of Public Health Sciences, Queen's University, Kingston, Ontario, Canada; 3 Western University, London, Ontario, Canada

**Keywords:** Pediatrics, child health, perceptions of health, generational lens, generational theory, mixed methods, qualitative research

## Abstract

Health perceptions adopted during childhood lay foundations for adult health trajectories and experiences. This study used a sequential mixed methods design to generate new evidence about child perceptions of health in two samples of Canadian children. A core qualitative study was followed by a complementary quantitative analysis to aid interpretation. Generational theory was used as a lens through which to interpret all data. Findings suggested that good health is perceived as customized and subjective. The strengths and liabilities of these perceptions are discussed, as well as implications for health promotion and prevention strategies. Through intentional consideration of the perspectives of this population group, this study makes both empirical and theoretical contributions to appreciating how cultural environments shape health perceptions.

The way children perceive and think about health influences the behavioral choices that they make (Hughner & Kleine, 2004). Further, health attitudes that are adopted during childhood and adolescence lay foundations for adult health trajectories and experiences (Felix et al., 2014; Williams, Holmbeck, & Greenley, 2002; Woodgate & Leach, 2010). Gaining a deeper understanding about the ways that children perceive health may have important implications for health promotion and primary prevention strategies including applied health policy.

Despite the potential importance of this topic, few existing studies have considered child perceptions and health conceptualizations as being distinct from those of adults (Aminzadeh et al., 2013; Wiens, Kyngas, & Polkki, 2014). Findings from this scant literature suggest that the criteria used by children to assess their health or their health behaviors appear to vary from those used by adults (Backett & Davison, 1992). Adults often evaluate health based on the presence or absence of self-limiting health problems (Piko & Bak, 2006), whereas for children, who are generally free of serious physical illness, health tends to be self-evaluated based upon more immediate behaviors and perceptions. The latter includes states of psychological well-being (Onyango-Ouma, Aargaard-Hansen, & Jensen, 2004), as well as engagement in physical activity (Piko & Bak, 2006), personal hygiene (Onyango-Ouma et al., 2004), adequate nutrition, abstaining from smoking, and good sleep habits (Pridmore & Bendelow, 1995).

Attitudes about health in young people are shaped not only by their age and stages of development, but by the prevailing cultural attitudes of their generation. While research using generational theory to understand attitudes of this current youth cohort has been applied to many fields, such as marketing (Lazarevic, 2012; Mobley, 2015), education (Holyoke & Larson, 2009; Kriegel, 2013), nursing (Foley, Myrick, & Yonge, 2012; Weingarten & Weingarten, 2013), and health care (Berkowitz & Schewe, 2011; Bickel & Brown, 2005), analogous empirical research has not been applied to childhood perceptions of health. This too may be an important gap in knowledge.

In this study, we used a sequential mixed methods design with a convergent mixed methods analysis (Teddlie & Tashakkori, 2009) to generate new evidence about child perceptions of health. We first conducted a core qualitative study and when unexpected findings emerged, we generated new hypotheses that could not be fully understood using the existing data. We then turned to quantitative methods to aid in their interpretation and used generational theory as a lens to reflect upon both sets of data. Together, these two analyses created a more in-depth understanding of the findings than either method could have done in isolation. This type of “emergent” mixed methods design generally occurs when a second approach (quantitative or qualitative) is added after the study is underway, because one method is found to be inadequate in and of itself, and to resolve unexpected study findings (Morse & Niehaus, 2009).

The core data source for the qualitative study was seven focus groups undertaken with a contemporary (2014) Ontario sample of 40 youth aged 12–15 years. This is a group of pre- and early adolescents who were born on the cusp of the 21st century. The secondary source, used to interpret and illustrate patterns observed qualitatively, is the 2010 Canadian Health Behaviour in School-aged Children study (HBSC). HBSC is a quantitative study and involves written health surveys conducted with students in classroom settings every 4 years, with a focus on health and health behaviors in the early adolescent years. In 2010 (HBSC Cycle 6), 25,912 adolescents were surveyed from across Canada. The national sample was stratified by province/territory, type of school board (public vs. separate), urban–rural geographic status, school population size, and language of instruction (French vs. English) and is considered to be representative nationally. In this article, the words child and adolescent are used interchangeably.

## What is generational theory?

A generation refers to a group of people who share experiences and perspectives because they were born at a similar time (Bickel & Brown, 2005). Each generation has characteristics that can be defined by the historical events, cultural and family norms, and political environments that shaped them during their developmental years (Holyoke & Larson, 2009). Generational theory is used to help explain how the time period of a person's birth affects his or her value systems. This in turn impacts how he or she interacts with people from other generations and the wider world (Mobley, 2015). Using generational theory as a lens for exploring child perceptions of health may provide new insights into current issues related to the health trajectories of today's young people.

The children in our qualitative sample were born between 1998 and 2002, and in our quantitative sample, between 1995 and 1999. Collectively, they represent the tail end of a generation that Howe and Strauss (2009) coined “the millennial generation” (born between 1989 and 2000) and that Crampton and Hodge (2006) refer to as Generation Y. They are also on the cusp of what Rosen (2011) and Wood et al., (2013) refer to as the “iGeneration,” which encompasses young people born in the 1990s and beyond. Both Rosen (2011) and Wood et al., (2013) note that the “i” represents not only the type of technologies with which these young people engage (iPhones, iPads, and iTunes) but also the highly individualized activities that these technologies make possible. In this study, we describe our participant population as “Millennials.” We recognize that there are limitations to this label, and that others would potentially apply a different name to this population.

Through intentional consideration of the perspectives of this population group, we aimed to contribute both empirically and theoretically to new understandings about the health perceptions of today's early adolescents. We sought to present a balanced view between youth perceptions of what constitutes good health and positive health behaviors, and a strong evidence base of scientific literature in the same area. Sometimes the two were in keeping with each other, and sometimes they were not. For the sake of long-term positive health trajectories in young people, both schools of thought are important. The mixed methods approach we undertook did enable us to embrace both points of view.

## QUAL/QUANT mixed methods study

### Overall method

**Figure 1:**
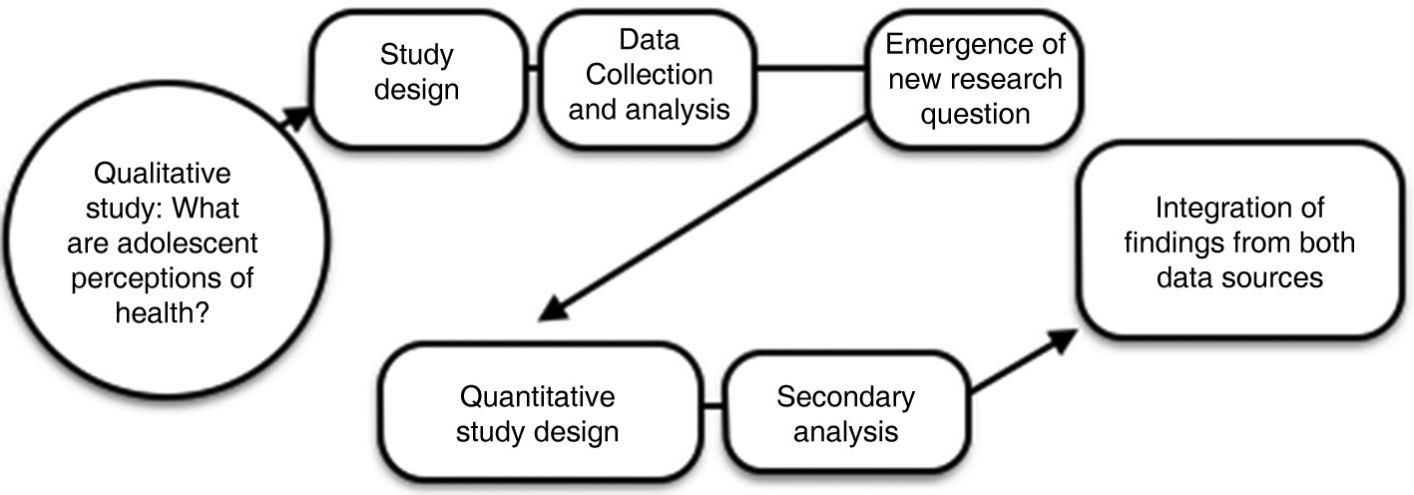
Mixed methods design.

This study used a sequential mixed method design. Findings from the initial 2014 core qualitative study were unexpected, and led to the formulation of questions we then posed of data that had been collected in 2010. The goal of this second quantitative strand was to provide further explanation for and interpretation of the initial qualitative findings. Final inferences were based on the results of both strands of enquiry (see Figure 1).

### Strand 1: qualitative

This study design followed the qualitative, constant comparative method of grounded theory (Glaser & Strauss, 1967), which entails that the project begins with an area of interest, rather than a preconceived theoretical perspective. We began with a general interest in youth perceptions of health, and data were generated as we asked open-ended questions related to this topic in focus groups of young people aged 11–15 years. During this process, the investigators were simultaneously involved in both data collection and analysis, and hence mid-collection analysis shaped subsequent data collection procedures. As theories began to emerge throughout initial coding and analyses, subsequent questions were then modified and became more focused. Data collected in the early stages of study permitted us to compile more evidence around emerging themes and questions. As interviewers, we focused questions iteratively in order to explore this theme more fully throughout subsequent data collection.

The use of the iterative strategies of constant comparison and memoing ensured that the emergent theory was grounded in the data.

### Participants

Participants were recruited using a purposeful, criterion-based approach. Participants who shared one or more criteria (including age, sex, rural/urban geographic status, and immigration status) were recruited. Homogenous groups of like participants (i.e., all boys, all girls, etc.) were formed in order to facilitate conversation, and diversity was achieved between groups. Our final sample included seven focus groups, who were selected from populations in Eastern Ontario (Hastings and Frontenac Counties), Northern Ontario (Greater Sudbury), Western Ontario (Bruce County) and the Greater Toronto Area. A total of 40 young people was involved (see Table I), with— four to seven participants in each group. All participants were recruited using “snowball” or “chain” sampling (Patton, 2001), by which well-situated people within communities (educators, community leaders, etc.) were asked to circulate information about the study to the parents of potential participants.

**Table I:** Demographic characteristics of the qualitative study population.

Gender	
Boys	13
Girls	27
Age (years)	
12–13	22
14–15	18
Size of community	
Large urban center	13
Medium size town	18
Rural locations	9
Total participants	40

### Data collection

Data collection was conducted within the seven focus groups. Open-ended questions were used to invite rich discussion from each group, and each group was also provided with the opportunity to engage in discussion about several standard definitions of health. After being provided several common definitions, they were asked: “Are there any words you would like to add or take away?” and “Do any of these definitions resonate with you more than others?”

Photo elicitation techniques were also used to elicit conversation (Harper, 2002). After the group had discussions about standard definitions of health, a series of small cardboard cards containing photographs were shown to participants, with hopes that these cards would elicit responses surrounding health. Photographs included well-known categories of health, including its physical, mental/emotional, social, and spiritual aspects as well as potential contextual determinants of health such as school environments, home environments, neighborhoods, and the larger environmental context of our planet. The photos were not meant to limit or strongly guide young people or the discussion, but rather were general enough to act as catalysts or prompts that aided participation and engagement. It was expected that participants would interpret the images subjectively, and bring their own ideas and experiences into the conversation (Flick, Von Kardordff, & Steinke, 2004). Participants were also given an opportunity for new and unanticipated themes to emerge. A sample question used for this activity was: “Do any of these cards help you to describe any aspects of being healthy or unhealthy?”

As discussion emerged, participants were invited to sort the cards that they had chosen into piles that reflected similar ideas about health. If participants thought that a card belonged in more than one category, they could request a duplicate. Once all the selected cards had been sorted into piles, participants were invited to work together to give a name to each pile. We then asked participants if there were any other ideas about health that belonged in these categories that were not represented by these pictures, or if there was any other groups that were required to represent the health of a person. Next, to facilitate ease of movement, the names of the piles were then written on translucent plastic circles. Participants were invited to organize the circles (representing the different ideas that had arisen during conversation) into a model that would “show us what health looks like all together in a person.” Participants were invited to discuss and work together and to move around during this activity.

Each group had many ideas about what good health is for people of their age, and each developed multiple categories to describe these distinct ideas. However, when we invited them to organize the categories into a model that would show us the health of a person, a strong and recurring feeling among the participants was that it would look different for everyone. This theme intrigued us early on, and iteratively, our methodology enabled us to probe this theme with more depth.

### Coding and analysis

A professional transcriber created a verbatim transcript of all focus group interviews. Line-by-line coding of the transcript then enabled us to identify each participant's and group's thought patterns, feelings, and ideas that emerged throughout the interviews. Initial, first level codes were generated directly from the text. This was done primarily by two researchers, who reviewed several transcripts in order to determine preliminary coding structures for organizing the data. Coded segments were then organized into categories. A third level of axial coding was then applied, which resulted in the identification of three, higher-level, more conceptual themes.

### Academic rigor

Throughout analysis, we employed tools of constant comparison, theoretical sensitivity, and triangulation. These techniques ensured that all codes, categories, and themes were grounded in the data. Constant comparison helped to ensure that each piece of data was considered in relation to previous and subsequent data, and that data was not fragmented but considered as a whole. Throughout the coding and analysis process, we also used theoretical memoing in order to record additional insights, questions, and themes. Multiple researchers engaged in critical dialogue around all aspects of data collection, coding, and analysis, which further enhanced the rigor of the study and minimized individual researcher bias.

### Ethical considerations

This study received ethics approval from the Queen's University Health Sciences & Affiliated Teaching Hospitals Research Ethics Board (approval number EPID-447-13 ROMEO/TRAQ #6011166). All parents provided written, informed consent and all participants gave written and verbal informed assent prior to participation in the study.

### Quantitative methods

Members of our research group are involved in the HBSC study. HBSC is a World Health Organization collaborative study of health and health behaviors in adolescents in 44 countries or regions (Currie, Gabhainn, & Godeau, 2009). Data compiled by HBSC in Canada offer a unique opportunity to examine health and its potential determinants in adolescent populations nationally. More specifically for this study, it provided a basis for a quantitative analysis that provided additional insight into the themes that emerged during our qualitative analysis.

### Study population and procedures

HBSC involves written health surveys conducted in classroom settings, with a focus on the early adolescent years (ages 11–15). It is administered every 4 years following a common international protocol. The 2009–2010 (Cycle 6) Canadian sample was stratified by province/territory, type of school board (public vs. separate), urban–rural geographic status, school population size, and language of instruction (French vs. English). Standardized population weights were generated to account for over- and under-sampling in specific regions as compared to the underlying provincial or territorial population demographic by grade and sex. Children from private schools, home schools, First Nations reserves, street youth, incarcerated youth, youth otherwise absent on the day of the survey, or youth not providing informed consent (explicit or implicit, as per school board customs) were excluded. Response rates were 11/13 (84.6%) at the province/territorial level, 436/765 (57.0%) at the level of schools (404 of whom answered an administrators’ questionnaire), and 26,078/33,868 (77.0%) at the student level. The HBSC study protocol was approved by the Queen's University General Research Ethics Board (GMISC-062-13; Romeo # 6010236).

### Measures

In response to the emerging qualitative theme that health, and particularly good health, is different for every young person, we examined available data from Cycle 6 of the Canadian HBSC. We did this to estimate the prevalence of known, major risk factors for chronic disease and injury among young people who also reported that they were in good or excellent health. This addressed the protective component of our study objectives, which was to identify perceptions of health that are not in keeping, long term, with behaviors that lead to positive health trajectories.

We first categorized HBSC respondents according to an item describing each child's rating of their general health status: Would you say your health is: (1) “Excellent”; (2) “Good”; (3) “Fair”; (4) “Poor” (Idler & Benyamini, 1997). The “excellent” and “good” categories were combined in subsequent analyses, as were the “fair” and “poor.”

We subsequently examined the reported prevalence of the following negative behaviors and health experiences: (1) low physical activity outside of school, in hours per week; (2) engagement in bullying of another student during the last couple of months; (3) smoking (daily, weekly, less than weekly, never); (4) binge drinking during the last 12 months, defined as having five or more alcoholic drinks on a single occasion among boys, and four or more among girls (weekly, monthly, less than monthly, never); (5) cannabis use during the past 3 days (four categories, “never” through “six or more times”); (6) feeling depressed or low during the last 6 months (five categories, “rarely or never” through “daily”); (7) feeling sad or low almost every day for 2 weeks in a row (“yes” or “no”). The idea here was to identify whether sizable proportions of young people who viewed their health as “good” or “excellent” were simultaneously having health experiences, or engaging in risk behaviors, that are known to be destructive to their health both acutely and chronically.

## Results from strand 1: qualitative

The study population for the qualitative strand is described in Table I. All participants (*n*=40) attended schools in the Ontario public school system and were fluent in English. All participants agreed to participate in the study, and all had the consent of a parent/guardian.

**Figure 2:**
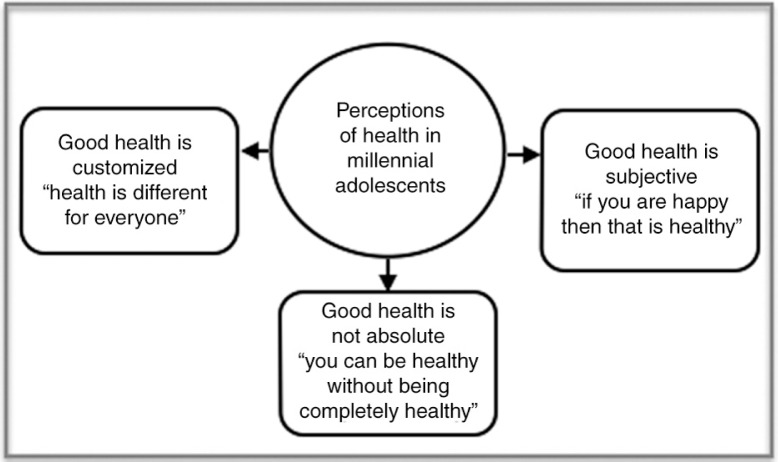
Perceptions of health in millennial adolescents.

Three overarching themes emerged that contributed to our understanding of how these participants perceived health. First, participants reported that good health is customized. This is a characterized by the idea that health is different for everyone. Second, participants reported that optimally, good health is subjective; how one feels about one's health state and behaviors is fundamental in determining if one is healthy or not. Third and finally, for these participants, good health is not absolute, a theme that one participant captured in the phrase “you can be healthy without always being healthy.” Reflections of each of these themes are provided below. The entire model is illustrated within Figure 2.

### Good health is customized: “Health is different for everyone”

The young people who participated in the qualitative component of our mixed methods study perceived health as “different for everyone.” The strength and consistency of this viewpoint was striking, and emerged between participants in individual groups and across focus groups. One participant emphasized the importance of this theme by identifying that “health is different for everyone” as the most important thing we had talked about in his focus group. Repeatedly, participants articulated that because each person is unique, each person has different needs, a different context, and different attitudes that fundamentally make their perception and experience of health customized.

One way that this theme emerged was in the way youth readily identified a diversity of behaviors, attitudes, and contexts that could be important to health in general. However, there was no consensus on what those aspects would be in a particular person. As one participant said, “Everyone has a different way of living” and so, “Different people need different things.” This theme of “different for everyone” related not only to what aspects contributed to health, but to the balance between the aspects. As one participant explained, this is because:

Different people need different amounts of each one. It depends on who you are and what you need to be healthy. Some people want more time with other people and that makes them feel healthier and then some people want more time by themselves and that makes them feel healthier. It depends on the person.

The young people reflected a very broad view of health, much bigger than purely physical health. When asked to rank which aspects of health were most important, the participants almost universally had trouble doing this. They were far more comfortable with a conception of health that allowed for the diversity of each person, and were unable to decide which aspect(s) of health would be more or less important. As one participant said: “It all matters but it is different for every person.”

What young people perceived to be healthy appeared to relate to a diversity of factors, including one's personality, context, interests, and body type. In several groups, it was suggested that being an introvert or an extrovert was important to consider in terms of health, because something that would be healthy socially for an extrovert may not be perceived as feeling healthy for an introvert. For example, when shown a picture of a young person sitting by himself on the grass, a participant said:

He is by himself so he is not being social. I am not sure if he is upset or if he is happy and just that is what makes him happy …. It could be that something happened and he is upset or that he just likes doing that and he is happy.

Repeatedly, participants observed that they, their peers, and people in general had different contexts, personalities and social, emotional, and physical needs, all of which contributed to the overall perception that health is different for everyone.

### Good health is subjective: “The state that you want to be in …”

For the young people in our study, good health was often described by criteria simply related to whether or not they felt that they were healthy. Illustratively, this subjectivity was reflected by the participant who told us that, “To be healthy you have to believe you are healthy and think of yourself and know that you are healthy.” This personal interpretation extended to evaluating health status, and was articulated in a way that was similar to the first theme (“health is different for everyone”). For example, one participant reported: “Certain people's healthy is not the same as other's healthy. This one's healthy could be different from my healthy.” Repeatedly, participants agreed that a primary factor in whether or not some behavior or health state was healthy or not was how that particular person felt about it.

One of the definitions to which participants were invited to respond was a standard World Health Organization (WHO, 1948) definition: “Health is a state of complete physical, mental, and social well-being and not merely an absence of disease.” When asked if any words stood out, one participant said:

I don't think that you have to be completely or perfectly physically, mentally, and socially happy with where you are. If you are halfway to being completely fine and happy with who you are … then that is alright. As long as you are happy with yourself, it doesn't matter how healthy you are.

The connection this participant made between feeling happy with who you are as a way of evaluating health status—or more precisely, that personal happiness causes health status not to matter—is a variation on the same theme of good health being subjective. Another participant said: “If you are happy then that is healthy but if you have no friends and are unhappy with that then that is not social well-being.” In other words, good health had little to do with measurable qualities, but rather was quite simply (in the words of the participant) “the state that you want to be in” or a state that you are happy or satisfied with.

### Good health is not absolute: “You can be healthy without being completely healthy”

This statement that “you can be healthy without being completely healthy” is representative of the way that many participants rejected any kind of absolute or ideal to describe health. One place this theme emerged was again in response to the WHO definition of health (WHO, 1948). In most groups, participants took exception to the word “complete.” One participant said: “No one is perfect. If you are saying someone is perfectly healthy or completely healthy then that is not true. No one can be absolutely and completely healthy.” At this point, another participant jumped in with her comment: “I would completely agree with this definition if the complete was not in there.” A third participant continued: “I think that complete should not be in there. As (anonymous) said: “You can't be completely and perfectly healthy.” There will be days when you just want to lie around or eat junk food …”

Another described a healthy person as “someone who balances diet and exercise but also enjoys food and just being lazy sometimes.” The need for balance and moderation in health attitudes and behaviors was clearly present, as evidenced by this longer excerpt describing an interaction between three participants in one group:

Participant 1: One of my cards … is a boy sitting on a couch watching TV eating cookies. I feel like this is an unhealthy thing to do because you are eating unhealthy food and lazing around. That is not necessarily true. For all we know … people who exercise a lot could spend some time in front of the TV as well. I don't think that you can classify it as an unhealthy lifestyle because he probably doesn't do it all the time.

One of the interviewers probed by asking: “What do you think about what he is doing in the picture right now?”

Participant 1: I wouldn't say that it is terribly unhealthy but it is not very healthy. I don't know … He could have been at a sports game or something and now he is trying to relax. After you do something strenuous it is nice to sit back and relax. I don't think that it is always healthy to be thinking: “Oh, I need to be healthy and eat more fruit.” I think that it is also healthy to say, “It is okay for me to eat cookies sometimes.”

Participant 2: I think that it would be unhealthy not to do that. I can see some people who are super healthy and get obsessed with it … If they always have to be perfect I think that would be really stressful and I don't think that would be healthy.

Participant 3: If he is always doing that then that is not healthy. But if he is just eating junk food and staring at a screen then that is not healthy. If you relax once in a while it is okay. Also, if you always have to be healthy you might, like (participant 1) said, be obsessed and become unhealthy.

The need for moderation in health behaviors was a key part of this theme.

## Strand 2

### Methods (quantitative)

In Table II, we present some examples of quantitative findings from Cycle 6 of the Canadian HBSC Study, which examine the health experiences of young people aged 11–15 years in Canada during 2009–2010 stratified according to their responses to a general health status measure. We examined the frequency of selected behaviors and health experiences according to whether young people viewed their health as “good” or “excellent,” or conversely “fair” or “poor.” Among those who reported that their health was “good” or “excellent”: (1) 38% reported that they exercise only 1 h or less per week outside of school hours; (2) 52% had engaged in bullying behaviors at least once over the past couple of months; (3) 8% smoked at least occasionally at present; (4) 44% engaged in binge drinking at least once during the past 12 months; (5) 15% used cannabis at least once in the past 30 days; (6) 24% felt “low or depressed” at least weekly during the past 6 months; (7) 17% had felt sad or low every day for 2 weeks in a row. While we are not promoting the idea that good health is necessarily devoid of all potentially negative behaviors at all times, each of these behaviors can be viewed as problematic in certain contexts, and these results suggests the need for caution in accepting the views of young people without question.

**Table II:** Engagement of young Canadians in health and risk behaviors in relation to their self-reported general health status, 2010 Canadian Health Behaviour in School-aged Children study.

	Self-reported health status			
	
	Good/excellent		Fair/poor	
		
	*N*	col%	*N*	col%
Exercise outside school, hours/week				
7 h or more	3377	16	213	5
4–6 h	4438	21	466	11
2–3 h	5238	25	885	21
About ½–1 h	6491	31	1857	45
None	1368	7	744	18
Taken part in bullying another student, past couple of months				
Never	9986	48	1546	37
Once or twice	7527	36	1582	38
2 or 3 times a month	1560	7	430	10
About once a week	865	4	260	6
Several times a week	876	4	329	8
Smoke tobacco at present				
Do not smoke	18,842	92	3188	79
Less than once a week	692	3	206	5
At least once a week	339	2	159	4
Every day	603	3	498	12
Binge drinking, last 12 months				
Never	4264	56	1010	49
Less than once a month	1461	19	385	19
Monthly	1369	18	425	20
Weekly	548	7	254	12
Cannabis use, last 30 days				
Never	6530	85	1504	72
1–2 times	447	6	179	9
3–5 times	225	3	84	4
6 or more times	494	6	314	15
Feeling low (depressed), last 6 months				
Rarely or never	10,961	53	1275	31
About every month	4718	23	900	22
About every week	2242	11	609	15
More than once a week	1642	8	701	17
About every day	1021	5	623	15
Feel sad or hopeless almost every day for 2 weeks or more				
Yes	3374	17	1418	37

## Discussion and integration of findings

The aim of this study was to contribute to an understanding of the way young people perceive health, and in turn better understand and inform avenues for positive health promotion for this population group. Our qualitative findings indicated that for participants in this study, their way of understanding health had three main components: good health is essentially “different for everyone,” good health is subjective, and good health does not have absolute criteria (in that there is room for “being unhealthy” in good health). The complementary quantitative strand suggested a need for some caution in accepting the subjective views of some young people about the positive nature of their health behaviors, because many young people who reported that their health was excellent also engaged in risk and other behaviors that could be considered unhealthy in many contexts. While we recognize that the qualitative strand of this study is limited in size (40 individuals) and scope (Ontario based sample), it led us to reflect on the way that using generational theory as a lens might shape perceptions of health in Millennial adolescents.

Generalizations about a particular group of people are subject to many exceptions based upon individual personalities and circumstances. However, a large and diverse body of literature offers characteristics that are often attributed to the Millennial generation as a whole and we have drawn upon that literature base throughout our discussion.

### Good health is “customized”

For the Millennial generation, customization is second nature (Becker, 2009). And for the iGeneration, while customization is usually facilitated by technology (for example, customized e-profiles that declare their interests, dating status and movie tastes), it is arguably even more strongly an inherent mindset that characterizes this generation (Rosen, 2010). From their education plans to their frappuccinos, customizing parts of the iGeneration's lives is done on a daily basis. Indeed, for both the Millennials and the iGeneration, most of life is customized much of the time. Wood et al., (2013) reflect on characteristics of this generation in the realm of healthcare, writing: “If music, television, advertising, and internet search engines can be customized and available in a click or a tap, then the same expectation will be placed upon healthcare” (p. 1). On reflection, it is no surprise then that our qualitative participants reported the expectation of a customized ideal of health, which was reflected over and over again by statements such as “Health is different for everyone.” These statements also reflect the trend in this population group toward inclusivity (Jonas-Dwyer & Pospisil, 2004). If health is different for everyone, then no one is left out, and each person's health state is viewed as normative.

This finding has many implications for health promotion initiatives. First, it is important to recognize the potential benefits of a customized perception of health: conceivably, it will serve as protective, for example, against the idea that everyone's body should look the same, and protect both boys and girls from idealizing a particular body type over any other. This health attitude validates and values different personality types and social needs (in particular, introverts and extroverts) and is quick to reject judgment of other people's health behaviors and ideas.

Second, this finding reinforces the need to consider the uniqueness and diversity of each person when talking with young people about health. This approach could create room for more meaningful conversations and teaching around health. This is important in terms of health promotion in that it suggests that the language young people are comfortable with in talking about health needs to make room for the individualized, customized cultural context in which they have been shaped. Clearly, a “one-size-fits-all” approach does not work for health promotion, particularly with Millennial youth, any more than it works for education, marketing, health care, or any other area of life.

However, this “one-size-does-not-fit-all approach to health,” along with its rejection of any kind of ideal measure (as observed quantitatively), does raise some concerns. For example, when the baseline measure of health becomes “whatever is right for me,” it is difficult in terms of health promotion to advocate for any “bettering” of health. It is not clear that young people perceive the line between individual preference and opinion, and the basic facts that some behaviors—such as smoking or drug use—are just not healthy for us as humans. In other words, if health is different for everyone, how can leaders in health promotion, health education, health care, parents, and teachers determine if something actually is—or is not—positive to health? If today's young people have adopted a customized model of health, it would be wise to invest serious efforts into understanding the strengths and limitations of using such an individualized approach, and to consider what can be done to draw from different and potentially equally useful and valid sources of knowledge about health for adolescent populations.

### Good health is subjective

It was the second finding, which related to one's subjective experience or opinion, that was initially most surprising to us, and we were unsure how to interpret it. Statements such as “To be healthy you have to believe you are healthy” were concerning because we could imagine a situation in which a young person believed a behavior to be healthy that was in contradiction to a base of scientific and biological evidence. Further, when participants said things like “As long as you are happy with your health, it doesn't matter how healthy you are,” we wondered if youth were ignoring the potential long-term trajectories associated with negative health choices in their adolescent years. In other words, young people are potentially overvaluing a subjective view of health (how they feel about their health) at the expense of a more objective view (which, for example, would support the idea that lack of physical activity is unhealthy). Both subjective and objective views of health are valuable. A problem emerges when one's subjective evaluation of health does not also take into account objective evidence.

Our questions led us to further study this theme quantitatively. The findings were striking, and highlight the disparities between subjective opinions about being healthy, and potential indicators of healthfulness that have been objectively measured. For example, as reported in Table II, among those who reported that their health was “good” or “excellent,” 38% did not meet daily recommended activity levels for Canadian adolescents, while over half had participated in negative bullying behaviors within the past couple of months, many reported risk-taking behaviors related to substance abuse, and nearly three-quarters of participants reported indicators of poor emotional health. This included 17% of those reporting good or excellent health reporting having felt sad or low every day for 2 weeks in a row, which is an initial screening item for a suicide ideation scale (Shaffer, Fisher, Lucas, Dulcan, & Schwab-Stone, 2000). All of these behaviors can be viewed as problematic in certain contexts, and our findings suggest the need for caution in accepting the views of young people without question, or as the only way of measuring their health states and behaviors.

Generational theory also offers a framework for interpreting these findings. Howe and Strauss (2007) suggest that Millennials, as a cohort, are characterized by a confidence that can easily become arrogance. Research also suggests that Millennials are a sheltered generation, and while they have been empowered to participate in family-based decisions their whole lives, they were also often protected from the consequences that can accompany decisions (Bourke & Mechler, 2010). The combination of confidence in one's own ideas, and not having had the experience of consequences resulting from poor decisions, may set Millennials up to be over-confident in their subjective experience of health without the maturity to consider other perspectives on health, and particularly on the long-term negative consequences of various health decisions.

Again, these qualitative and quantitative findings have important implications for health promotion. While subjective well-being has been demonstrated to be a powerful indicator of health (Idler & Benyamini, 1997), many studies have shown that such reports can be inaccurate. For example, Goodman, Hinden, and Khandelwal (2000) showed that teens were generally inaccurate when reporting obesity; self-reported abnormal sleep patterns were also inconsistent with actual sleep patterns in children (Bauer & Blunden, 2008). The potential danger reflected in our findings is that young people will choose to only consider their own subjective experiences (reflected in the quote “if you are happy then it's healthy”) while ignoring other perspectives and forms of evidence.

### Good health is not absolute

The third qualitative theme was captured by the quote “You can be healthy without being completely healthy.” This suggested a desire for moderation and balance, which we recognized as a potentially healthy attitude. Through the lens of generational theory, we are perhaps observing the desire for balance that is representative of this group in many areas of life (Howe & Strauss, 2007).

The recognition of moderation and balance as important is potentially key for helping Millennial youth to achieve good health. Without the stress of being “perfectly healthy or completely healthy,” which can lead to becoming “obsessed” and thus “unhealthy,” perhaps the Millennial generation is positioned to find a positive middle way. At its best, this third finding could be a foundation for developing a customized model of health that was also based on scientific evidence and not only subjective feelings.

Indeed, both our qualitative and quantitative findings tell a similar story: while many young people feel that their own knowledge about and perceptions of health related to positive health, their perceptions may be in contradiction to a strong evidence base that would suggest otherwise. A balanced view holds that while it is important to listen to and value the subjective ideas of young people, more objective ways of evaluating health and health behaviors are also important. Adults must not lose sight of their own important role in teaching young people health lessons that are based on evidence, even when this involves challenging young people's views.

While we began this study by examining adolescent perceptions of health, we were also provided with a mirror into the deeper ideas and beliefs that characterize this generation. This has enabled us to draw attention to the way that generational trends impact perceptions of health in this population group. This is important because adolescence is a critical developmental period, and ideas about health that are formed during this time lay foundations for adult health trajectories (Felix et al., 2014; Williams et al., 2002; Woodgate & Leach, 2010).

When developing health promotion strategy for Millennial populations, balance and moderation should be considered as important components. For example, when a healthy desire for customized approaches to health is balanced with a strong evidence base of health research, millennial youth may reap the benefits of a model of health that is “different for everyone” but at the same time, is rooted in solid evidence. Likewise, a balanced approach will involve valuing young people's ideas, and yet challenging views that are in contradiction with evidence based information about long-term consequences of poor health choices.

## Limitations and strengths

Limitations of this study warrant comment. The qualitative strand was limited geographically to the province of Ontario, Canada, and hence may not be generalizable across populations and places. Because focus groups may be dominated by one or two personalities, some children may not have felt comfortable sharing their own opinions honestly. This danger was, however, mitigated somewhat by the experienced facilitators who were trained to build rapport and enable each person's voice to be heard. Finally, all participants were first approached about participation through their parents. This may have led to a positive bias in that children with a positive parent/child relationship may have been more likely to agree to participation and children with poor parent/child relationships may not have been approached or may have declined participation.

The quantitative strand of this study also has limitations. Responses were by self-report, and there may be variation in the way that participants understood and provided reports for individual questions. Such misclassification, if random between groups, would typically cause attenuation of effects. Further, the HBSC does not include young people in private or home school situations, on First Nations reserves, incarcerated youth or youth who are not at school on the day the survey is conducted. The HBSC is conducted in a cross-sectional manner, and therefore it is difficult to establish the temporal aspects required to make causal inferences.

Our study also has many strengths. Along with providing further understanding about how health is perceived, we provided the opportunity for young people to have a voice in our program of research. Participants in the focus groups were articulate and engaged. The resultant data are textured, deep and nuanced, and observations were honestly and generously shared. Because our study focused on questions that had significant practical value, the thoughts and ideas that participants shared were rooted in their real-life experiences. Participants came from a variety of age groups, and represented the views of both genders and a diversity of school, socio-economic and family experiences. Though two-thirds of the focus group participants were girls, the themes that emerged in our focus group that was exclusively boys were consistent with results from the full sample. This study gives voice to a sample of young people and in this way, makes a significant contribution to conversations around adolescent health. The quantitative strand of this study had additional strengths. Findings from this analysis should be representative of adolescent health experiences nationally. The sample was large, diverse, and the questions used have been subject to piloting and validity checks across many countries and cultures (Freeman et al., 2011). The findings, while simple, provide clear and convincing evidence that the perceptions of young people do not always coincide with what is known about the origins and nature of healthy child experiences. Our mixed methods design was also a strength of the study overall. Qualitative findings provided rich contextual understanding of youth perceptions of health, and enabled us to include a genuine youth voice. The quantitative strand then enabled us to observe an important trend in a large sample, generalizable to a larger population. Together, the integration of our findings enabled better understanding about what we had observed in the results of both studies.

## Conclusions

Qualitative findings from this study suggested that for these adolescent participants, health is perceived as being “different for everyone.” While it was agreed that health has many different components, there was no consensus on what these components should be. Further, participants perceived that “you don't have to be healthy to be healthy,” and that indeed, a number of perceived “unhealthy behaviors” actually made an important contribution in the wider picture of a person's health. Quantitatively, we observed a percentage of young people who identified their health as excellent or very good, and yet who simultaneously reported behaviors that have been identified as risk factors for poor health and that are not in keeping with common objectives measure or conceptualizations of good health behaviors.

Using generational theory as a lens through which to intentionally consider the perspectives of this population group, this study makes both empirical and theoretical contributions to appreciating how adolescent perceptions of health may vary from those of adult populations, and further, how cultural environments shape health perceptions. Understanding perceptions about health held by Millennial adolescents will help adults to engage with this population group about important health issues on their own terms, and in ways that connect with their lived reality and contribute to the development of effective health promotion strategies. For the sake of long-term positive health trajectories in young people, when children's perceptions of their own health states and health behaviors are in contradiction to a known body of evidence, more objective ways of understanding and measuring health can be helpful in gaining a balanced evaluation of a child's health.
